# Supplementary material for: Long-Term Outcomes of T1 Colorectal Cancer after Endoscopic Resection
Source: J Clin Med. 2020 Jul 31;9(8):2451. doi: 10.3390/jcm9082451 (PMC7464364; doi:10.3390/jcm9082451)
Supplement: Supplementary file 1 [file jcm-09-02451-s001.pdf]

Supplementary Table S1. Comparison of clinicopathological characteristics in the ASR subgroup of the NC-ER group according to recurrence.

	**Recurrence N (%) (Overall N =3)**	**No recurrence N (%) (Overall N =114)**	***p*-value**
Age (years), mean ± SD	68.0 ± 4.4	62.7 ± 9.5	0.160
Sex, n (%)			>0.999
Male	2 (66.7)	77 (67.5)	
Female	1 (33.3)	37 (32.5)	
Location, n (%)			>0.999
Colon	3 (100)	101 (88.6)	
Rectum	0 (0)	13 (11.4)	
Size (mm), mean ± SD	14.7 ± 6.8	17.0 ± 5.6	0.555
Macroscopic type, n (%)			0.059
Sessile	0 (0)	66 (57.9)	
Flat	0 (0)	15 (13.2)	
LST-G	0 (0)	6 (5.3)	
LST-NG	3 (100)	27 (23.7)	
Resection method, n (%)			>0.999
ESD	3 (100)	103 (90.4)	
EMR	0 (0)	11 (9.6)	
Pathology			
Well and moderately differentiated	3 (100)	114 (100)	
Submucosal invasion depth	1667 ± 1155	2612 ± 1286	0.291
Lymphovascular invasion			>0.999
positive	0 (0)	14 (10.6)	
negative	3 (100)	100 (89.4)	
Margin			0.558
positive	2 (66.7)	43 (37.7)	
negative	1 (33.3)	71 (62.3)	

ASR, additional surgical resection; EMR, endoscopic mucosal resection; ESD; endoscopic submucosal dissection; LST-G, laterally spreading tumor–granular type; LST-NG, laterally spreading tumor–non-granular type; NC-ER, non-curative endoscopic resection.

Supplementary Table S2. Risk factors for recurrences in the ASR subgroup of the NC-ER group.

	No. of patients	No. of events	Univariate			Multivariate		
			**HR**	**95% CI**	**p-value**	**HR**	**95% CI**	***p*-value**
Age								
≥65	27	1	1.739	0.158–19.186	0.651	1.107	0.066–18.632	0.944
<65	90	2	1					
Sex								
Male	79	2	1					
Female	38	1	1.014	0.092–11.182	0.991	1.112	0.073–17.020	0.939
Size (mm)								
≥15	54	2	2.062	0.187–22.745	0.555	4.938	0.289–84.259	0.270
<15	63	1	1					
Submucosal invasion depth (μm)								
≥2500	56	1	0.528	0.048–5.828	0.603	0.238	0.013–4.347	0.333
<2500	61	2	1					
Lymphovascular invasion								
Positive	14	0	0.000	0.000–inf	0.999	0.000	0.000–inf	0.999
Negative	103	3	1					
Margin								
Positive	45	2	2.992	0.271–32.995	0.371	1.531	0.110–21.424	0.751
Negative	72	1	1					

ASR, additional surgical resection; CI, confidence interval; HR, hazard ratio; NC-ER, non-curative endoscopic resection; ASR, additional surgical resection.
